# Case report: Diagnosis and treatment of pulmonary choristoma in a newborn calf

**DOI:** 10.3389/fvets.2023.1257329

**Published:** 2024-02-01

**Authors:** Noriyo Usaki, Takeshi Tsuka, Midori Hatanaka, Yuji Sunden, Aoi Imamura, Takehito Morita

**Affiliations:** ^1^Hyogo Prefectural Federation Agricultural Mutual Aid Association, Hyogo, Japan; ^2^Department of Veterinary Clinical Medicine, Joint Department of Veterinary Medicine, Faculty of Agriculture, Tottori University, Tottori, Japan

**Keywords:** bronchi-like structures, calf, Doppler ultrasonography, feeding arterial vessel, pulmonary choristoma

## Abstract

A 4-day-old female Holstein calf presented with a large-sized, protruding mass in its back, at birth. Radiography identified the deformed spinous process in the second and third lumbar vertebras, suggesting spina bifida. Ultrasonography of the back mass revealed anechoic bronchi-like structures and large vessels with rich blood flow running parallel within the homogenous echogenic mass’s parenchyma. Doppler ultrasonography also revealed pulsatile vessels entering into the deeper side of the transverse process of the lumbar vertebras at the right-sided base of the protruding mass. These imaging results were helpful for surgical planning, in which a large arterial vessel was sutured at the right-sided mass’s base, followed by resection of the mass itself. The mass’s resection could be carried out according to the planned surgical procedure, though its invasion was too deep to be resected completely. Histopathology for the resected specimens revealed that the mass mainly had lung-tissue-like structures comprised of bronchi-, bronchiole- and alveoli-like structures, and large vessels, allowing the diagnosis of pulmonary choristoma. Doppler ultrasonography could contribute to the differentiation between the bronchi-like tubular structure and the large arterial vessels on the same images, aiding diagnosis of this disease.

## Introduction

Choristoma is an embryonic developmental anomaly where histologically normal tissues from various organs form in different anatomical positions to their origins (1–4). Choristoma origins have reportedly included mammary, striated muscular, endometrial and müllerian tissues in human patients (1, 2, 5–8).

Pulmonary choristoma (PC) is a congenital anomaly characterized by the formation of mass lesions due to reduplication during embryonic development or derived from supernumerary lung buds (9). This disease is referred to as an ectopic or accessory lung, bronchopulmonary foregut malformation, and extralobar pulmonary sequestration (9, 10). Bronchogenic cysts are also etiologically associated with pulmonary sequestration (11). Lung tissue heterotopia also resembles PC in terms of lung-tissue-derived mass formation but has different histological findings, e.g., no formation of mesothelial cell layers (12). In human medicine, pulmonary sequestration is frequently used to differentiate this extralobar type from the intralobar type predominantly found within the lung structures or the visceral pleura (9, 10, 13). Extralobar pulmonary sequestration accounts for 14%–26% of human cases of pulmonary sequestration (10, 14). In the veterinary literature, the congenital anomalies derived from lung tissues have been referred to by other names, including pulmonary sequestration (15, 16), an ectopic or accessory lung (17, 18), bronchopulmonary foregut malformation (19) and PC (3, 4, 9, 20). A retrospective study using 28 bovine cases with PCs confirmed the subcutaneous involvements of these lesions in approximately 32% of animals, compared with approximately 68% of animals with intra-abdominal and intra-thoracic lesions (21). The other previous bovine report described the prevalence of these disease’s thoracic, abdominal and subcutaneous involvements as approximately 10, 50 and 40%, respectively (16). Additionally, the anatomical positions where the subcutaneous lesions have been affected are the head, neck, shoulder, chest, back, and anus (4, 9, 16, 19, 22, 23).

Skeletal deformity is minorly found concurrently in human or animal cases, with choristomas originating from various organs as well as the lung (6, 13, 14). Compared with the lower prevalence (14%) of the intralobar type of pulmonary sequestration accompanied with other anomalies, half of human patients with the extralobar type (PC) had various concurrent anomalies, accounting for 4% in involvements of skeletal deformities (14). Regarding congenital skeletal deformity associated with PC, two previous newborn calves had wide frontal or parietal bone defects associated with masses protruding from their head (3, 16). Congenital vertebral abnormalities were also found in the previous bovine cases involving PCs in their backs (22, 23). Although the cause of the formation of spina bifida associated with PC is not fully known, as identified in the present case, subcutaneous formation of the congenital masses within the back, such as lipoma, choristoma, teratoma, and hamartoma, prior to neural tube closure can contribute to the formation of the spina bifida occulta in human patients (1, 24). Common with the previous two animals with PCs on their heads, the ectopic structures may function as mechanical obstacles that induce skull fusion failure during the developmental period (3, 16). Alternatively, the vertebral defects may be simply one of the common embryogenic anomalies that occur during the same fetal period as PCs; previous reports described the association of vertebral defects due to pulmonary hypoplasia and bronchogenic cysts (15, 25–27). Two previous human cases with intralobar pulmonary sequestration had congenital defects, including intravertebral fusion in their cervical vertebras, separate from the locations of this disease (28). Congenital neuro-vertebral and foregut anomalies may be synchronized, resulting in neural arch defects (such as spina bifida and hemivertebrae) and formation of pulmonary sequestration due to developing at the same embryological stages (28, 29). In bovine fetuses, the formation of supernumerary lung buds, as an origin of PC, may occur between days 30 to 50 of gestation (9, 26). Multiple concurrent anomalies such as ectopia cordis, extra-hindlimb, and rudimentary humerus have also been found in a previous calf with a PC mass on its back (17). Thus, when diagnosing PC, it is necessary to try to detect the associated skeletal deformities, including spina bifida (30).

A protruding, swollen back mass was the common macroscopic characteristic in the previous bovine cases with PCs on their backs (22, 23, 30). Thus, the macroscopic observation allows easy detection of this disease when supported by palpation (11, 12, 18, 19, 22, 23). However, a differential diagnosis cannot be made based on the swollen backs, due to the shared macroscopic appearances of the cutaneous or subcutaneous mass lesions such as spina bifida cystica, cellulitis, abscess, tumors (e.g., lipoma) and tumor-like masses (e.g., hamartoma) in newborn or younger animals (4, 24, 25, 30–37). Additionally, the macroscopic and palpation examinations cannot provide pathological findings deeper than the superficial masses, such as the degree of invasion or destruction of the underlying structures and vasculature. In particular, it is very difficult to identify congenital skeletal deformity in such examinations (22, 23, 30).

Radiography and computed tomography (CT) were the imaging modalities used for diagnosing and therapeutic decision-making in the previous bovine cases with various types of choristomas in multiple sites (15, 16, 18–20, 23, 26). The uses of radiography and CT are helpful for evaluating the mass’s invasion for the underlying structures and the concurrent involvements, including skeletal deformity (15, 16, 18–20, 23, 26). However, ultrasonography has not been used as a diagnostic tool, despite one previous bovine report describing the use of ultrasonography for diagnosing a bronchogenic cyst (11). In human medicine, ultrasonography is a valuable imaging modality (despite its inferior to contrast CT) for evaluating the intra-mass abnormal vasculature that indicates intralobar or extralobar pulmonary sequestration (14, 38, 39).

The present report describes the clinical use of radiography and ultrasonography for a newborn calf presenting with PC in its back and the surgical procedure for resectioning this mass. The diagnostic efficacy of two imaging modalities, in particular, the two specific ultrasonographic signs, is discussed based on the results from previous human and animal reports.

## Case presentation

**Figure 1:**
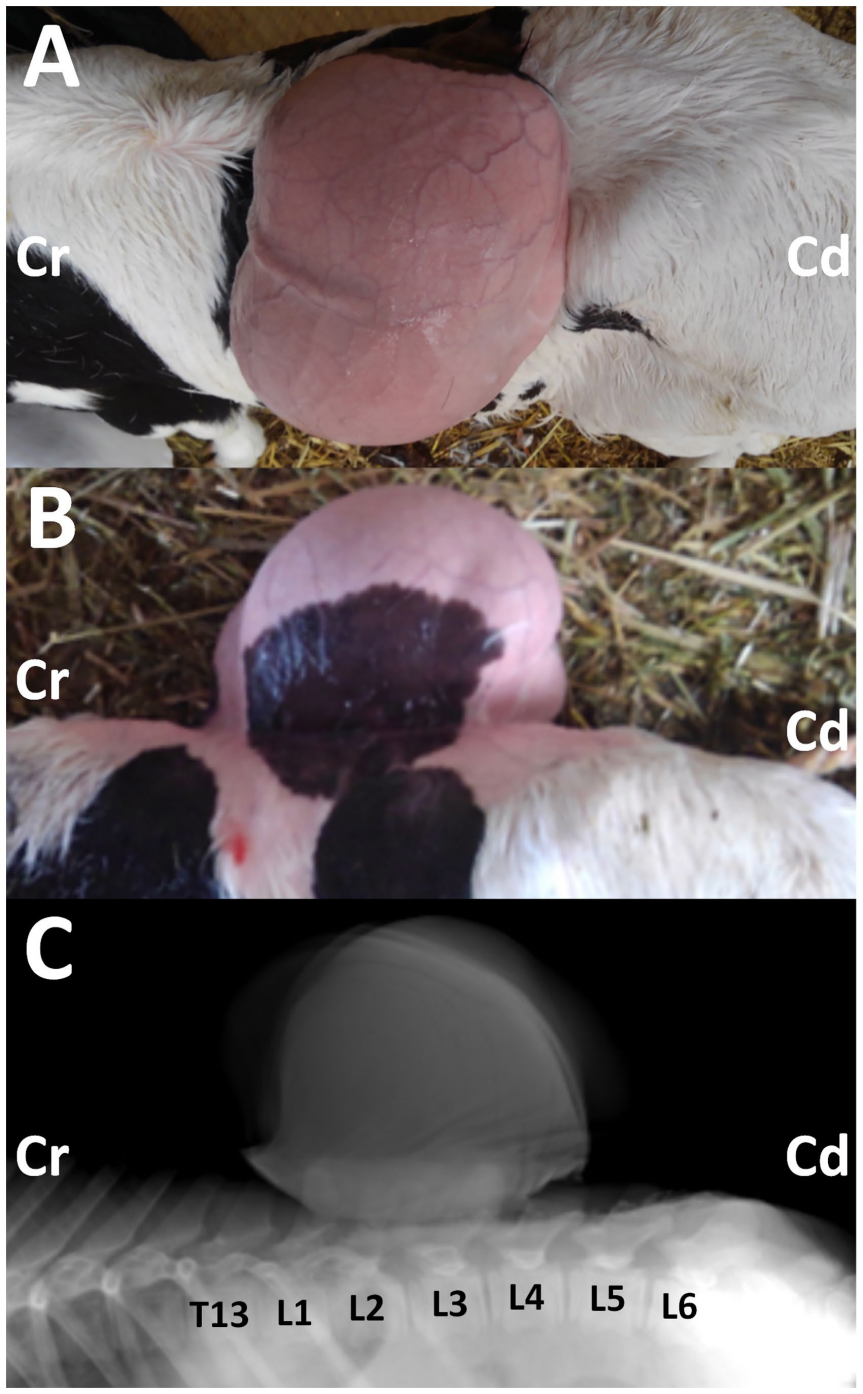
Photos from the dorsal **(A)** and lateral **(B)** views of the 3-day-old female Holstein calf with the protruding mass in its back, when taken after clipping on day 14. Cr: cranial; Cd: caudal. Lateral radiograph **(C)** showing the mass located on the level between the first and fourth lumbar vertebras (L1 and L4). The deformed spinous process is evident in the second and third lumbar vertebras (L2 and L3), compared with the radiopaque shapes of the spinous process in the thirteen thoracic vertebrae (T13), and the first, fourth, fifth and sixth lumbar vertebrae (L1, L4, L5, and L6). Cr: cranial; Cd: caudal.

A female Holstein calf weighing approximately 40 kg was born with owner‘s delivery assistance. At that time, owner noticed formation of a large-sized swelling in its back locating at the level between thirteen thoracic and fourth lumbar vertebras (Figures 1A,B). At 4 days old, this calf had a normal physical condition, a good appetite, and was active and drinking normally. This case could walk normally without neurological weakness in its hindlimbs. The back swelling was caused by the subcutaneous formation of the mass, which measured 20 cm, 30 cm, and 20 cm in its cranio-caudal length, width, and height, respectively. Palpation identified that a rounded, soft mass lesion was present subcutaneously, with separation from the skin at the transition level between the thoracic and lumbar vertebras. The subcutaneous mass was anatomically connected with soft tissue structures underneath it.

Radiography was performed in the lateral recumbent position under sedation, with an intramuscular injection of xylazine hydrochloride (0.2 mg/kg). Lateral radiography identified a large-sized, smooth mass located close and dorsally to the second and third lumbar vertebras (Figure 1C). The mass was homogenously radiopaque in the center, lined by the higher radiopaque contour. At the base of the mass, irregular, heterogenous radiopaque structures were observed to overlap with the spinous process of the lumbar vertebras. Within the overlapping region, deformation in the spinous process was evident in the second and third lumbar vertebras. This finding suggested spina bifida, despite the unclearness caused by the overlapped mass.

**Figure 2:**
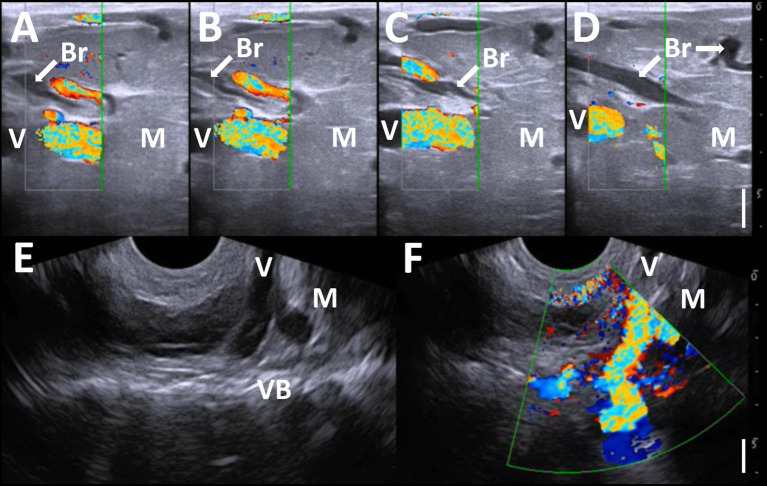
Doppler ultrasonograms **(A–D)** of the mass, when scanning by the cranial-caudal moving of a 10.0 MHz linear transducer. Anechoic bronchi-like structures (Br) and the large vessels with rich blood flow (V) run within the homogenous echogenic mass’s parenchymas (M), separated by the hyperechoic septal structures. Ultrasonogram **(E)** and Doppler ultrasonogram **(F)** showing the bifurcation part of the large vessel (V) in the ventral margin of the protruding mass (M) at the levels of the lumbar vertebral body (VB), when scanning the right-sided base of the mass using a 3.5 MHz microconvex transducer. Bar: 1 cm.

Ultrasonography was performed using a portable ultrasound device (MyLabOne VET, Esaote Corporation, Genova, Italy). After the calf was clipped, sprayed with alcohol, and had ultrasound gel applied, a 10.0 MHz linear transducer was applied to the swollen back of the non-sedated animal in its standing position. This revealed a larger 8–10-mm diameter vessel running within the homogenous echogenic parenchyma at approximately 3 cm under the skin’s surface (Figures 2A–D). Additionally, 2–5-mm diameter vessels were also evident at the parenchyma proximal to the large vessel. The tubular structures were seen to run alongside these vessels within the spaces between them. These structures had anechoic lumens outlined by 1–2 mm thick hyperechoic wall structures, suggesting a bronchi-like structure. The gentle handling of the applied transducer to follow the large vessel identified several anechoic tubular structures, keeping their parallel position while wrapping themselves around the vessel’s branches. These tubular structures seemed to have anatomical communication, though anatomical communication between the large vessel and the branches could not be demonstrated on the same ultrasonograms. Doppler ultrasonography identified pulsate blood flows within the lumens of the large and small vessels, suggesting that intra-mass vessels were arterial vessels. The large vessel could be visualized as running toward the body, when scanning the right-sided base of the mass using a 3.5 MHz microconvex transducer (Figures 2E,F). This vessel had a diameter of over 1 cm at this level and was separated into two parts. On the ultrasonogram, the cranial part of the bifurcation of the vessels ran straight toward the hyperechoic structures, showing the transverse process of the lumbar vertebras, despite the vessel’s running not being evident at the deeper level. Doppler ultrasonography on the same scanning position revealed that this vessel had a pulsatile blood flow and ran deeper than the lumbar vertebras’ transverse process. However, the main arterial vessel could not be detected as the end of this vessel. Based on these ultrasonographic findings, this mass was suspected to be a pulmonary choristoma. With this information, the surgical procedure could be planned: suturing the large vessel at the level of the mass’s base should occur before removing the mass, to ligature the interception of completely arterial blood flows.

**Figure 3:**
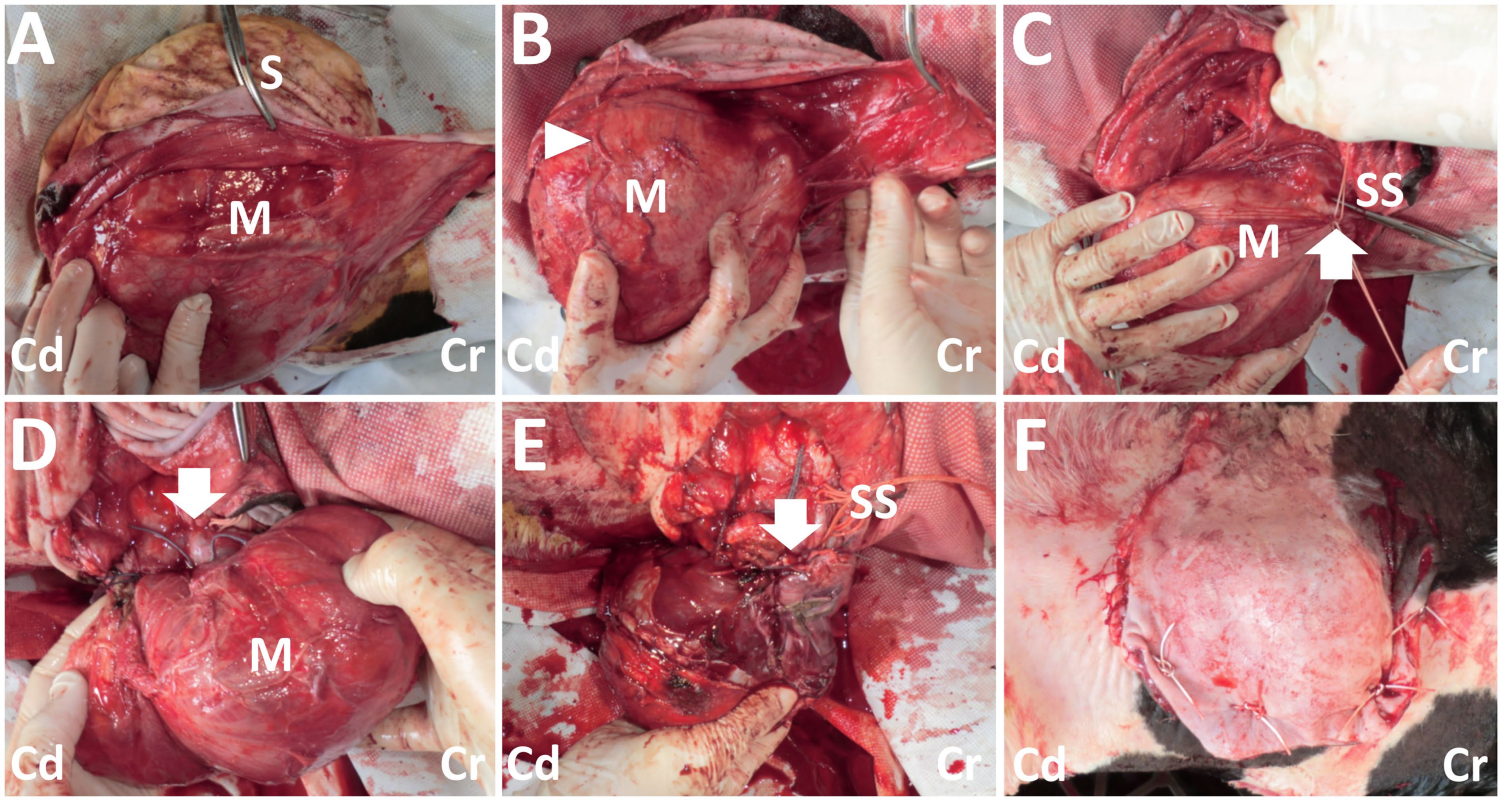
Photos of the surgical procedure for resection of the back mass. **(A)** A dark-red mass (M) is exposed when the incised skin (S) is dissected bluntly. **(B)** The large, tortuous vessels (arrowhead) are evident on the surface of the mass (M). **(C)** Two braided silk sutures (SS) are set around the part of the vessel’s entry (arrow) to the mass (M). **(D)** The part of the vessel’s entry (arrow) to the mass (M) is sutured. **(E)** Resection of the mass is carried out, while the stalk-like structure of the mass’s base is sutured with braided silk suture material (SS). Arrow shows the resected part. **(F)** The macroscopic views after skin suturing. Cr: Cranial; Cd: Caudal.

On day 14, the calf was anesthetized with an intramuscular injection of xylazine hydrochloride solution (0.2 mg/kg, xylazine injection 2% Fujita, Fujita Pharmaceutical. Co., Ltd., Tokyo, Japan). Local anesthesia was made by subcutaneous injection of a 50 mL solution of procaine hydrochloride (Enpro injection KS, Kyoritsu Seiyaku Co. Ltd., Tokyo, Japan) around the swollen region. A 20 cm skin incision was made along the margin of the right-sided base of the mass. Blunt dissection of the subcutaneous tissues soon allowed exposure of a dark-red surface of the mass (Figure 3A). On the surface of the mass, several 2–5-mm diameter tortuous vessels were macroscopically detected (Figure 3B). At the cranial parts of the mass’s base, entering within the deeper structures, an approximately 1-cm diameter pulsate vessel could be detected. Blunt dissection of the large vessel from the mass’s surface was impossible as thickened, membranous structures tightly covered it. The route of this vessel could be detected macroscopically when lifting up the mass via surgical opening; this vessel made a detour around the median area of the mass, ran caudally, and then turned right. This vessel finally entered deeper within the space between the transverse process of the first and second lumbar vertebras. At the part of the vessel’s entry, this vessel was sutured firmly using braided silk suture material (Nescosuture, Alfresa Pharma Co. Ltd., Osaka, Japan) at positions proximal and distal to the planned incision part, as close to it as possible (Figures 3C,D). This vessel could be cut without arterial bleeding. Subsequently, each small vessel located on the mass’s surface was also sutured. After the complete cutting of the main vessels, blunt dissection for the transitional part between the mass and the underlying soft tissue structures allowed exposure of the mass base. This 7-cm diameter, stalk-like base entered deep into the space between the dorsal muscular layers. The end of the stalk-like structure could not be detected when palpated along its route by the operator’s fingers. Thus, suturing was made using a silk suture material for the stalk-like structure of the mass, as deep as possible. The incision was made at the proximal part than the suturing. Three-time suturing-incision procedures could allow the mass resection at the location of 1–2 cm under the skin’s surface (Figure 3E). The resection part was closed by buried suturing using an absorbable suture material (Covidien Polysorb Sutures USP 2–0, Covidien Japan Co., Ltd., Tokyo, Japan), followed by skin suturing (Figure 3F). The animal was treated with a five-day course of intramuscular penicillin–streptomycin mixed solution (Mycillin Sol, Tamura Pharmaceutical Co. Ltd., Osaka, Japan), followed by a five-day subcutaneous administration of enrofloxacin solution (Baytril 5%, Bayer Yakuhin Ltd., Osaka, Japan). No complication was evident in the area of the surgical wound, despite it being slightly swollen and hard for several weeks postoperatively. However, the animal exhibited progressive hindlimb paralysis coincident with gradual development of its back swelling, resulting in approximately 1 cm thick protrusion accompanying with purulent discharge between 3 and 4 months after surgery. Thus, the re-grown mass was surgically removed on day 177 (163 postoperative days). However, re-operation could not allow complete improvement of hindlimb paralysis, despite there was no swelling in the surgical wound. The animal was euthanized by intravenous injection of potassium chloride (approximately 2 nmol/kg) until auscultation identification of cardiac arrest under deep anesthesia with intravenous infections of Xylazine hydrochloride (1 mg/kg) and propofol (10 mg/kg) on day 196 (182 postoperative days), as per the owner’s request. Necropsy revealed formation of the fibromatous, capsular mass enveloping the purulent materials that could not be removed on re-operation because of extending deeply to the location of the underlying lumbar vertebras. Additionally, the defected spinous process of second lumbar vertebra, identified as a spina bifida, could be macroscopically observed as the route on mass-associated compression to the spinal cord.

**Figure 4:**
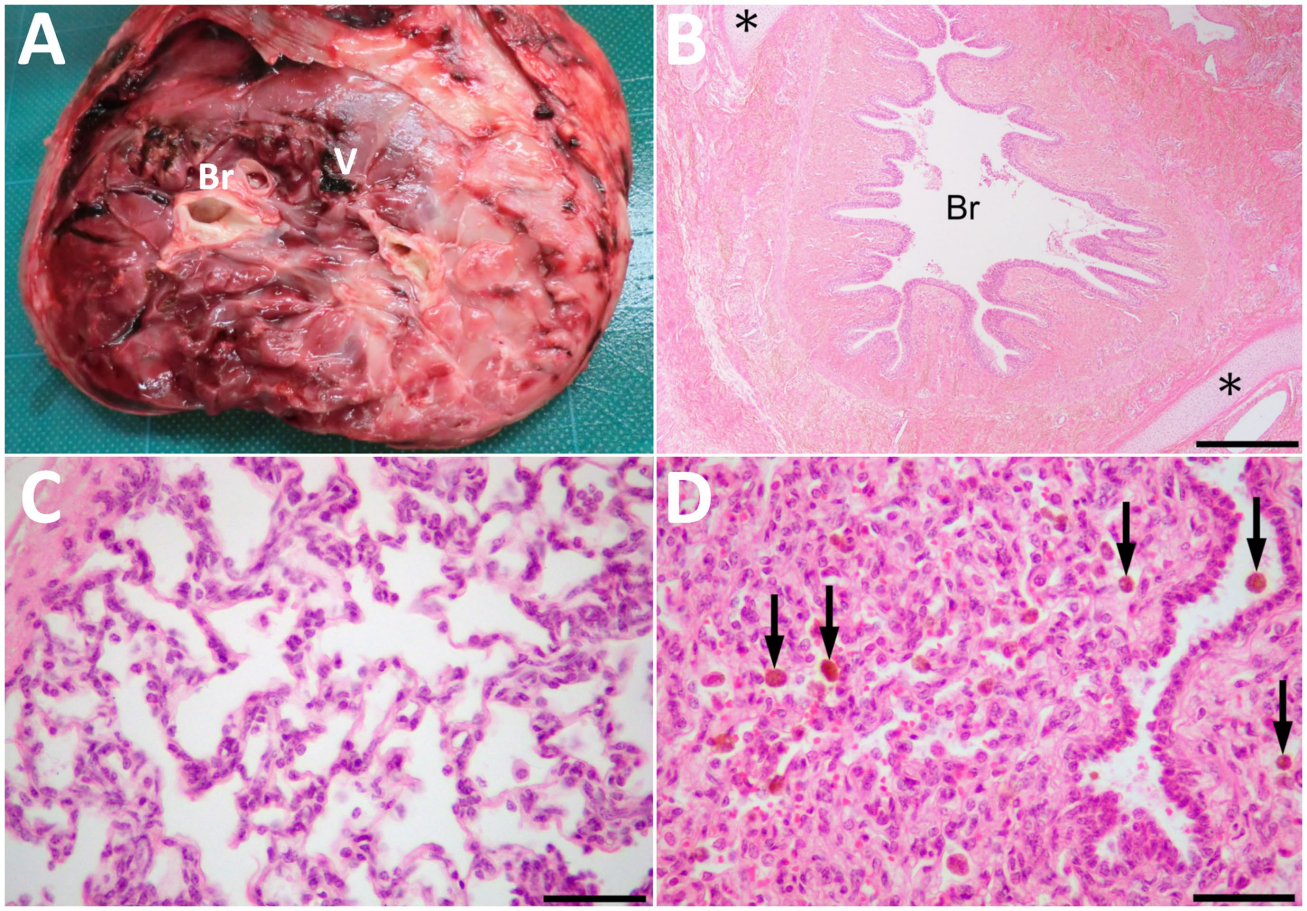
The cut surface of the resected mass **(A)** showing the bronchi-like structures (Br) and the large vessel (V) in the center of the parenchyma, including mixtures of hard and spongiform structures. **(B)** Histopathologically, the mass comprised of lung tissues, including bronchioles (Br) and collagenous tissues. Cartilage tissues (asterisks) locate around the bronchioles. HE. Bar, 500 μm. **(C)** Alveolus-like structures similar to pulmonary alveoli in a magnified view of the mass. Fibrous tissue similar to pleura is on the left upper of the photo. HE. **(D)** The compressed area is similar to atelectatic lung tissue. Arrows indicate hemosiderin-laden macrophages within the alveoli and bronchiole. HE. Bars are 50 μm in **C** and **D**.

Macroscopically, the mass had a dark-red-colored, irregular outer surface, covering the internal structures entirely, when removed surgically on day 14 (Figure 4A). The mass parenchyma included mixtures of hard and spongiform structures. Within the mass parenchyma was a rich network of blood vessels with various lumen diameters. The larger vessels measured approximately 1 cm. These vessels were gathered toward the mass’s center, forming the vessel’s plexus in the cut surface of the mass’s base. Multiple bronchi-like structures were also evident within the mass parenchyma. These bronchi-like structures strongly resembled the tracheobronchial tree within the normal lung. The contents were mostly empty (including air) within the lumens formed by the circumferential surrounding, cartilage-like walls of these structures. The bronchi-like structures had a lumens diameter > 1 cm in the cut surface of the mass’s base.

Histopathology of the resected mass contained the branching bronchi-like structures with a single layer of columnar ciliated epithelial cells (Figure 4B). Deposition of collagenous fibers, middle- to large-sized arteries, cartilage tissues and pulmonary alveoli-like structures were also found within the mass (Figure 4C). Alveolar spaces were irregularly expanded with mild infiltration of macrophages and neutrophils. Those alveolar structures were lobulated by fibrous tissues, and compressed lobules were similar to atelectatic lung tissue. Congestion, hemorrhage, hemosiderin-laden macrophages, edema and fibrin deposition were also noted in these areas (Figure 4D). Other histological findings of the mass were granulation tissue formation, fibrous tissues similar to interlobular connective tissues in lungs and pleura, adipose tissues and a few peripheral nerve bundles. Based on these histological findings, the mass was diagnosed as pulmonary choristoma.

## Discussion

This report included the diagnostic efficacy of two imaging modalities, radiography and ultrasonography, in the present case. In previous bovine cases involving PCs in their neck and chest, the clinical uses of radiography aimed to evaluate the degree of the mass’s invasion into the underlying soft tissues and thoracic cavity (11, 15, 19). In a previous bovine case with an intrathoracic lesion, chest radiography could identify the clear, smooth line between the anechoic and echogenic contents; these represented gas and fluid, respectively, dorsally and ventrally within the cystic cavity enveloped by the mass’s capsular structure (26). Skull radiography for a previous bovine case with a head lesion demonstrated the fissure’s defect in the affected frontal bone (16). These radiographic findings were valuable for deciding surgical intervention (11, 15, 16, 26). Based on the clinical data in the previous human and bovine cases presenting with various types of choristomas in their backs, radiography is helpful for identifying the lack of the radiolucent shapes of the spinous process, allowing suspicion of spina bifida at the lumbar vertebras (1, 5, 23, 24). Previous data support the concurrent involvement of spina bifida in the present case, combined with the unfavorable outcome due to progressive hindlimb paresis. In the present case’s lateral radiograph, abnormality in the spinous process was not clearly detectable due to the overlapping of the mass and the peripheral vertebras (23). Thus, ventrodorsal or dorsoventral radiographs should be taken for the affected lumbar region, as these are preferable for evaluating malformation of the spinous process compared with lateral radiographs (37).

The use of radiography makes it difficult to differentiate PC from other subcutaneous soft tissue masses (40). This mass could be demonstrated on radiographs as a soft tissue opacity, identical to the present case (11). Soft tissue opacity was the common radiographic appearance of the back masses in the previous animals with spina bifida cystica, hamartoma and infiltrating lipoma (31, 34). Contrast radiography seems to be less diagnostic aid because it did not enhance the mass’s radiopacity (15).

Ultrasonography is superior to radiography in terms of the visibility of soft tissue structures, despite its poor visibility of bone structures. Ultrasonography is frequently used to observe superficial swelling such as abscesses, hematomas, hernias, bursitis, and tumors (32–36, 41, 42). The echotexture of PC is a well-defined or irregular homogeneous echogenic solid mass (39, 43, 44). The common ultrasonographic appearance is easily distinguishable from those of abscesses, hematomas, and bursitis demonstrated as the capsular masses, including variably echogenic contents with or without the echogenic septal structures (35, 36, 41, 42). Spina bifida cystica, which has the same macroscopic appearance, can appear as a cystic mass filled with anechoic or hypoechoic fluids on the ultrasonograms of swollen backs. The echogenic mass of the brain or spinal cord can be seen in the fluids if meningoencephalocele or myelomeningocele develops (45). Bronchogenic cysts must also be distinguished from PC: they appear ultrasonographically as a mixed echogenic cystic mass, despite the same embryologic origin (11, 40). The ultrasonographic findings of abscesses and tumors or tumor-like masses demonstrate a mixed consistency and echogenicity, but ultrasonography does not present enough evidence to differentiate these from PC (32–35). Based on the results obtained from the present calf with a PC back mass, as no reports discussed using ultrasonography for a similar disease, the two ultrasonographic signs seem useful for differentiating this disease from the solid masses.

One specific ultrasonographic sign is the tortuous, large vessels with pulsatile blood flow within the mass. This sign has already been observed in previous human reports, where ultrasonography revealed that intra-mass abnormal vessels had large lumen diameters measuring 1–12 mm (14, 29, 38, 43, 46). Arterial supply to PC is acquired via the large abnormal vessels derived from systemic vessels, most commonly including the descending thoracic and abdominal aorta (4, 9, 13, 14, 21, 27, 30). Unfortunately, despite the origin of blood flow being unknown in the present case, the intra-mass vessels might be derived from the abdominal aorta. This suggestion is based on their route out of the mass, which then entered the abdominal cavity via the space between the transverse processes of the first and second vertebras. Additional evidence of arterial supply could be obtained from the pulsatile blood flows into the tortuous, large vessels, as viewed by Doppler ultrasonogram of the mass. Intra-mass vasculatures that supplied arterial flows from the systemic vessels were the common macroscopic findings observed when removing the back and chest masses of the previously-reported bovine cases (19, 22, 23). The previous uses of Doppler ultrasonography for hamartoma and infiltrative lipoma revealed that the masses were avascular (32, 34). Thus, the Doppler ultrasonographic findings of the intra-mass abnormal vasculatures can be effectively used as significant evidence to differentiate this disease from various subcutaneous mass lesions (14, 39, 40, 44). In most human cases, however, the abnormal vessels are too small to be detected on Doppler ultrasonograms (39, 44). In bovine practice, Doppler ultrasonography is used to detect abnormal vessels as a PC indicator because the vessels commonly measure centimeters in diameter within large masses. To detect the major vessels providing intra-mass blood flow and feeding vessels within the mass, contrast radiography or fluoroscopy may show the blood flow route from the main vessels toward the mass via the tortuous abnormal vessels (13, 47).

Another ultrasonographic sign is the intra-mass anechoic tubular structures, which run along the abnormal vessels, representing bronchi-like structures. This sign was not evident in the previous uses of ultrasonography in human cases with intralobar or extralobar pulmonary sequestration (38, 40, 43, 46). Difficulty in visualization of abnormal bronchial structures within the mass may be dependent on the smaller diameter of small intra-mass feeding vessels, limiting their visibility (39, 44). There may be macroscopic or histopathological differences between the human and bovine specimens in the abnormal tubular structures comprised of bronchi-bronchioles-alveoli-like structures within the masses. In previous human cases, the bronchioles-like structures seemed predominantly present. In previous human cases, the bronchioles-like structures seemed predominantly present (40, 44, 46). On the other hand, within the specimens obtained from the previous bovine cases, the branches of the bronchi-bronchioles-alveoli-like structures were observed within the parenchymal structures (15, 16, 18, 21). The bronchi-like structures had macroscopically- detectable tubular sizes and were microscopically lined by the various epithelial walls, mostly hypoplastic (15, 16, 18, 21). Ultrasonographic visibility of the intra-mass bronchial structures may depend on the degree of hypoplasia of the bronchi-like structures within the mass. Based on our results, Doppler ultrasonography is required to distinguish between the bronchi-like tubular structures and the feeding vessels, as these two structures run together within the mass.

If the present case has been examined with CT, this advanced diagnostic imaging modality could have yielded the comprehensive information obtained from radiography and ultrasonography. CT would have offered superior visibility of osseous and soft tissue structures. The valuable CT evidence would have included the relative anatomical location between the soft-tissue back mass and the underlying skeletal structures in the present case. In a calf with a skull PC mass, CT has been previously used to detect an absence of communication between the subarachnoid space and this disease (20). In another calf with intrathoracic pulmonary sequestration, the mass’s dimension and relation to the surrounding lung structures were evident (26). Based on previous human medicine, CT angiography can show the intra-mass abnormal vasculatures and the formation of large branches derived from the major artery, such as the descending and abdominal aorta (7, 10, 12, 39, 44, 47). The venous drainage, such as the pulmonary vein toward the mass, is not always evident with CT angiography involving the chest cavity, despite the venous vessel of diameters ≥10 mm being detectable (44). The present case had a single large feeding vessel to supply blood flow into its back mass. The single type of feeding vessel is predominantly detected in human cases, compared with the multiple types, reportedly accounting for 15–20% of pulmonary sequestration (47).

The preoperative uses of imaging modality are very helpful for surgical planning for subcutaneous masses (11, 15, 16, 18, 20, 26, 42). No radiographic findings of the mass’s invasiveness to the thoracic cavity could support the surgical decision to intervene for neck and chest PC masses (11, 15). Preoperative uses of radiography and CT could contribute to the complete resection of intra-thoracic PC mass by thoracotomy, resulting in a favorable outcome (26). In the present case, ultrasonography contributed to the preoperative planning of the surgical route to approach the mass and large feeding vessel. Based on Doppler ultrasonogram of the pulsatile blood flow of the feeding vessel, it was found that this vessel required suturing prior to surgical resection of the mass itself to prevent arterial bleeding during surgery. However, percutaneous scanning on the surface of the extremely large PC back mass could not show the interface between the mass and the underlying structures, preventing ultrasonographic evidence from predicting whether the mass could be completely resected (42). Unfortunately, the present case had an unfavorable outcome five months after surgery, in which progressive hindlimb paresis developed, resulting in euthanasia. PCs can contribute to the formation of the underlying skeletal deformity when involved subcutaneously (16, 23, 30). Resection of the extremely large, subcutaneous PC mass may be challenging for such bovine cases. Re-growth of the remaining mass can compress the brain and spinal cord via defects in the skull’s fissure and the spinous process, induced by surgery-associated inflammation and infection. Surgery for one previous calf with a PC back mass resulted in progressive hindlimb paresis, followed by euthanasia between one month postoperatively (23). Another previous calf with a PC skull mass died suddenly on the way back to the farm on the day of surgery (16).

## Conclusion

Combination use of radiography and ultrasonography is required to diagnose PC in the back, accompanying spina bifida in the underlying vertebra. This disease can be differentiated from the other back subcutaneous masses based on the ultrasonographic signs of tortuous, large vessels with pulsate blood flow and bronchi-like structures running side by side within the mass.

## Data availability statement

The raw data supporting the conclusions of this article will be made available by the authors, without undue reservation.

## Ethics statement

The animal studies were approved by Tottori University Regulations on Animal Experiments. The studies were conducted in accordance with the local legislation and institutional requirements. Written informed consent was obtained from the owners for the participation of their animals in this study.

## Author contributions

NU: Data curation, Investigation, Writing – original draft. TT: Conceptualization, Data curation, Supervision, Writing – original draft, Writing – review & editing. MH: Data curation, Investigation, Writing – original draft. YS: Data curation, Investigation, Writing – original draft. AI: Data curation, Investigation, Writing – original draft. TM: Data curation, Investigation, Writing – original draft.

## Supplementary material

Supplementary Movie S1 On Doppler ultrasonogram, when scanning by the cranial-caudal moving of a 10.0 MHz linear transducer, several 5–10-mm vessels are shown between zero to five seconds of this movie. These vessels run tortuously and show pulsatile blood flows within the Doppler-scan window. From five seconds onwards in this movie, anechoic tubular structures run along the pulsate vessels within the mass parenchyma in the same image.

Supplementary Movie S2 Ultrasonography identifies the bifurcation part of a 10-mm diameter vessel in the ventral margin of the protruding mass at the levels of the transverse process of the lumbar vertebra when scanning the right-sided base of the mass using a 3.5 MHz microconvex transducer. From 11 seconds onwards in this movie, Doppler ultrasonography shows the pulsatile blood flow in this vessel, the cranial part of bifurcation that runs deeper than the transverse process of the lumbar vertebra. However, the entry of this vessel to the systemic artery is not evident.
